# Lack of Relationship between Fibrosis-Related Biomarkers and Cardiac Magnetic Resonance-Assessed Replacement and Interstitial Fibrosis in Dilated Cardiomyopathy

**DOI:** 10.3390/cells10061295

**Published:** 2021-05-23

**Authors:** Paweł Rubiś, Ewa Dziewięcka, Magdalena Szymańska, Robert Banyś, Małgorzata Urbańczyk-Zawadzka, Maciej Krupiński, Małgorzata Mielnik, Sylwia Wiśniowska-Śmiałek, Aleksandra Karabinowska, Piotr Podolec, Mateusz Winiarczyk, Matylda Gliniak, Monika Kaciczak, Jan Robak, Arman Karapetyan, Ewa Wypasek

**Affiliations:** 1 Department of Cardiac and Vascular Diseases, Jagiellonian University Medical College, John Paul II Hospital, Pradnicka St. 80, 31-202 Krakow, Poland; ewa@dziewiecka.pl (E.D.); swisniowskasmialek@gmail.com (S.W.-Ś.); akarabinowska@gmail.com (A.K.); ppodolec@interia.pl (P.P.); 2 Department of Molecular Biology, John Paul II Hospital, Prądnicka St. 80, 31-202 Krakow, Poland; m.szymanska@szpitaljp2.krakow.pl (M.S.); e.wypasek@szpitaljp2.krakow.pl (E.W.); 3 Department of Radiology, John Paul II Hospital, Pradnicka Street 80, 31-202 Krakow, Poland; p.banys@szpitaljp2.krakow.pl (R.B.); m.urbanczyk@szpitaljp2.krakow.pl (M.U.-Z.); m.krupinski@szpitaljp2.krakow.pl (M.K.); m.mielnik@szpitaljp2.krakow.pl (M.M.); 4 Students’ Scientific Group at Department of Cardiac and Vascular Diseases, Jagiellonian University Collegium Medicum, John Paul II Hospital, 31-202 Krakow, Poland; winiarczyk.mateusz@gmail.com (M.W.); matylda.gliniak@gmail.com (M.G.); monikakaciczak@gmail.com (M.K.); jahu114@gmail.com (J.R.); arman47k@gmail.com (A.K.); 5 Faculty of Medicine and Health Sciences, Andrzej Frycz Modrzewski Cracow University, 31-202 Krakow, Poland

**Keywords:** dilated cardiomyopathy, replacement fibrosis, interstitial fibrosis, biomarkers, collagen

## Abstract

The relationship between circulating fibrosis-related molecules and magnetic resonance-assessed cardiac fibrosis in dilated cardiomyopathy (DCM) is poorly understood. To compare circulating biomarkers between DCM patients with high and low fibrosis burdens, we performed a prospective, single-center, observational study. The study population was composed of 100 DCM patients (87 male, mean age 45.2 ± 11.8 years, mean ejection fraction 29.7% ± 10.1%). Replacement fibrosis was quantified by means of late gadolinium enhancement (LGE), whereas interstitial fibrosis was assessed via extracellular volume (ECV). Plasma concentrations of cardiotrophin-1, growth differentiation factor-15, platelet-derived growth factor, procollagen I C-terminal propeptide, procollagen III N-terminal propeptide, and C-terminal telopeptide of type I collagen were measured. There were 44% patients with LGE and the median ECV was 27.7%. None of analyzed fibrosis serum biomarkers were associated with the LGE or ECV, whereas NT-proBNP was independently associated with both LGE and ECV, and troponin T was associated with ECV. None of the circulating fibrosis markers differentiated between DCM patients with and without replacement fibrosis, or patients stratified according to median ECV. However, cardiac-specific markers, such as NT-proBNP and hs-TnT, were associated with fibrosis. Levels of circulating markers of fibrosis seem to have no utility in the diagnosis and monitoring of cardiac fibrosis in DCM.

## 1. Introduction

In healthy individuals, cardiac the extracellular matrix (ECM) stays in a state of homeostasis, i.e., anabolic processes, such as the synthesis of ECM components, are in balance with catabolism (the degradation of collagens) [1,2]. Optimal cardiac morphology and function are determined solely by the quantity and quality of myocytes and the collagen and other ECM compounds surrounding them.

After coronary artery disease (CAD) and hypertension, dilated cardiomyopathy (DCM) is the third most common cause of heart failure (HF) [3]. Unlike other causes, DCM typically occurs in adolescence and young adults and thus becomes a life-long and potentially fatal disease. During the course of DCM, the heart undergoes profound structural changes; these include left and right ventricular (LV, RV) enlargement, wall thinning, changes from an elliptical geometry towards a spherical one, the development of pulmonary hypertension, and fibrosis of the ECM [4].

Cardiac fibrosis is one of the hallmarks of DCM and is typically observed in 40–60% of cases [5,6]. Fibrosis significantly contributes to the progression of HF symptoms and functional impairment; it also increases the risk of re-entrant arrhythmias, leading to increased morbidity and mortality in DCM [7]. Strictly speaking, there are two types of cardiac fibrosis—replacement and interstitial—of which the etiology, pathology, and clinical meaning differ from one another [1]. Replacement (or reparative) fibrosis is a consequence of myocardial injury, is usually focal, and mainly functions to preserve the integrity and function of the heart after injury. In contrast, interstitial (or reactive) fibrosis develops in response to general processes, such as hypertension, valve diseases, inflammation, or genetic mutations, and is typically widespread and maladaptive [1,2,8].

Endomyocardial biopsy (EMB) and the microscopic assessment of cardiac samples have long been considered the gold standard in fibrosis assessment [9]. On the other hand, non-invasive methods, particularly with cardiac magnetic resonance (CMR), which allow for an accurate assessment of replacement and interstitial fibrosis, are currently building momentum [10]. During the process of fibrosis, ECM proteins and/or fibrosis by-products are released into the bloodstream. The measurement of circulating fibrosis-related molecules can provide insights into end-organ fibrosis [11]. Nevertheless, the source of measurable circulating molecules, and their relation with specific organ fibrosis, are not clear, and are the subject of ongoing research.

The primary hypothesis underlying this study was that patients with high levels of replacement and/or interstitial fibrosis would have significantly different levels (i.e., either increased or decreased) of circulating biomarkers in comparison to those with no (or with a minimal) cardiac fibrosis burden. Therefore, the aim of this study was to compare the circulating levels of biomarkers between DCM patients with high and low cardiac fibrosis burdens.

## 2. Materials and Methods

### 2.1. Study Population

This was a prospective, single-center observational study. A total of 102 consecutive patients with a diagnosis of DCM with stable HF symptoms (NYHA I-III class) over at least 2 weeks were recruited for the study. DCM was diagnosed following the current recommendations of the European Society of Cardiology (ESC), based on (1) morphological criteria (LV dilation detected via echocardiogram or CMR), (2) functional criteria (impaired LV ejection fraction, LVEF < 45%), as well as the exclusion of (a) significant CAD (>50% luminal stenosis) as detected by coronary catheterization or computed tomography coronary angiography, (b) primary heart valve disease, (c) congenital heart disease, and (d) severe arterial hypertension [12,13,14]. All patients underwent a detailed diagnostic work-up, including laboratory tests, transthoracic echocardiography, and CMR. All echocardiographic examinations were performed on commercially available equipment by experienced echocardiographers, in line with the recent joint American and European recommendations [15]. All patients received guideline-approved optimal HF therapy [13]. The control group consisted of 27 age- and sex-matched healthy volunteers, who underwent laboratory tests, including measurements of circulating fibrosis-related markers and detailed echocardiography.

### 2.2. Cardiac Magnetic Resonance 

CMR exams were conducted on a 3.0-T scanner (Magnetom Skyra, Siemens, Erlangen, Germany) at the time of inclusion. The CMR studies were analyzed with Syngo.VIA software version VB 40 (Siemens, Erlangen, Germany) in compliance with the post-processing guidelines sourced from the Society of Cardiovascular Magnetic Resonance [10]. Steady-state free precession cine images were acquired in consecutive short-axis slices covering the LV and three long-axis (2-, 3-, and 4-chamber) slices. The CMR protocol encompassed cine CMR, native and post-contrast T1 mapping, and late gadolinium enhancement (LGE) imaging.

#### 2.2.1. Assessment of Replacement Fibrosis

Consecutive short-axis LGE images covering the LV were obtained approximately 15 min after the intravenous injection of 0.1 mmol/kg of body weight of gadolinium-based contrast agent. Fibrosis was deemed to be present if LGE was noted on both short- and orthogonal long-axis LGE images. The quantitative analysis of the extent of LGE was assessed using a 5-standard-deviations threshold on consecutive short-axis slices, and was calculated as a percentage of the total LV mass (%LGE) [10].

#### 2.2.2. Assessment of Interstitial Fibrosis

T1 mapping was implemented using the Modified Look-Locker Inversion-Recovery (MOLLI) sequence before, and 15 min after, gadolinium-based contrast agent injection. The following typical MOLLI sequence tfi2Dl parameters were used: breath-hold TR/TE of 281/1.1 ms, slice thickness of 8 mm, FOV from 320 × 260 mm^2^, matrix of 144 × 256 pixels, and a flip angle of 35°. Native and post-contrast T1-values were determined by drawing regions of interest (ROI) in every segment of the basal, mid-ventricular, and apical slice according to the AHA 16-segment model, as well as in the center of the LV cavity for the measurement of T1 blood pools. ROIs were drawn in the mid-wall region of the myocardial segments, and were copied between native and post-contrast T1 maps. Segments with artifacts were excluded. The global native and post-contrast T1 times were calculated as the means of all segments. Extracellular volume (ECV) was calculated according to the established formula [10]:

ECV = ((1/(postcontrast T1) − 1/(native T1))/(1/(blood postcontrast T1)) − 1/(blood native T1))*(1 − Hct)

A blood sample was obtained on the day of scanning to measure hematocrit.

### 2.3. Laboratory Measurements 

Blood was collected using EDTA as an anticoagulant and the samples were then centrifuged (1600× *g*) for 10 min at 4 °C. The plasma was transferred to centrifuge tubes and stored at −20 °C until the time of the analysis. Plasma concentrations of cardiotrophin-1 (CT-1), growth/differentiation factor-15 (GDF-15), and platelet-derived growth factor subunit B (PDGF-BB) were determined using the Nori Human Enzyme-Linked Immunosorbent Assay (ELISA) Kit according to the manufacturer’s protocol (Genorise Scientific, Inc.; Glen Mills, PA, USA). The assay sensitivity of the Nori Human Cardiotrophin-1 ELISA Kit was 12 pg/mL, and the detection range was 62–4000 pg/mL. The sensitivity of the Nori Human GDF-15 and the PDGF-BB ELISA Kit was 5 pg/mL, and there was a measurable range of 25–1600 pg/mL for both tests. The intra-assay and inter-assay coefficients of variation for the tested biomarkers were <7%. Quantification of collagen type I and III synthesis markers in plasma samples was performed using an ELISA assay in accordance with the manufacturer’s directions (Bioassay Technology Laboratory, Shanghai, China). The level of sensitivity of the assays was 2.26 ng/mL for procollagen I C-terminal propeptide (PICP), and 2.52 ng/L for procollagen III N-terminal propeptide (PIIINP), respectively. The detection range for the human carboxyterminal propeptide of type I procollagen ELISA kit was 5–1500 ng/mL, whereas for the human N-terminal procollagen III propeptide ELISA kit, the detection range was 5–2000 ng/L. The intra-assay and inter-assay coefficients of variation, respectively, were <8% and <9% for PICP and <7% and <10% for PIIINP. The determination of the degradation products of C-terminal telopeptides of type I collagen (CTPI) in human plasma was conducted using Serum CrossLaps ELISA (Immunodiagnostic Systems Limited, Boldon, UK). The detection limit was 0.020 ng/mL, and the detection range was between 0.020 and 3.380 ng/mL. Both intra- and inter-assay imprecision values were <10%.

### 2.4. Statistical Analysis

All values are presented as mean ± standard deviations or percentage (counts). The Shapiro–Wilk test was used for the analysis of the normal distribution of quantitative variables. The comparisons of the continuous variables were conducted with a *t*-test or Mann–Whitney test when appropriate, and the comparisons of the qualitative parameters were carried out using the Chi^2^ test. The correlation analyses were conducted based on a Pearson correlation when normality was confirmed, otherwise the Spearman rank correlation was employed. All parameters (presented in Table 1 and Table 2) differentiating LGE present and absent groups (with *p*-values < 0.10), or associated with ECV (*p* < 0.05) were included in the regression analyses. The associations between the analyzed parameters and LGE were analyzed with uni- and multivariate logistic regression methods; the analysis of ECV made use of linear regression models. Redundant parameters (correlated with other predictors with R > 0.5) were not included in multivariate linear and logistic regression models. Areas under the receiver operating curve (ROC), referred to as AUC, were calculated to assess the cut-off values of NT-proBNP for the presence of LGE. All results were considered statistically significant when the *p*-value was <0.05. The Statistica package, version 13.0 (StatSoft, TIBCO Software Inc., Palo Alto, CA, USA), was used for the statistical analysis.

## 3. Results

### 3.1. Baseline Characteristics

The final study population consisted of 100 DCM patients with complete data. There were 44% patients with LGE and the median ECV was 27.7%. Patients were divided according to the presence of LGE and their median value of ECV. The comparison of the baseline parameters between DCM patients with and without LGE, and between groups from lower and upper ECV strata, is presented in Table 1. Patients with LGE differed from those without LGE in terms of the prevalence of hypertension, chronic obstructive pulmonary disease, diastolic blood pressure, QRS width, thickness of interventricular septum, left atrial area, E/E’ ratio, LV mass, native T1 times, ECV, hematocrit, white blood count, uric acid, and loop diuretic daily dosages. Patients from the lower ECV strata differed from those in the upper ECV strata in terms of the following: age, atrial fibrillation, ventricular arrhythmia, distance in the 6-min walk test, left and right atrial areas, prevalence of at least moderate mitral and tricuspid regurgitation, LGE extent, native and post-contrast T1 times, blood levels of uric acid and cholesterol, and loop diuretic daily dosages. Although ECV correlated with LGE extent, the correlation was weak (R = 0.37, *p* < 0.001).

### 3.2. Comparisons of Biomarkers between DCM Patients, Stratified According to LGE and ECV

In terms of standard cardiac biomarkers, both NT-proBNP and hsTnT differed between patients with and without LGE, as well as between patients with smaller and larger ECV (Table 2). However, none of analyzed fibrosis serum biomarkers were associated with LGE or ECV (in the correlation analysis between the fibrosis biomarkers present and ECV; all *p*-values were >0.05). Levels of all circulating fibrosis markers were significantly different between DCM patients and control subjects (Supplementary Table S1).

### 3.3. Associations between LGE and Biomarkers

Out of all the parameters differentiating patients with and without LGE, only NT-proBNP and LV mass were found to be independently associated with the presence of LGE (Table 3). NT-proBNP was strongly associated with LGE, with an AUC of 0.70 (95% CI 0.6–0.8; *p* < 0.001) and a cut-off value of 635 pg/mL (sensitivity of 71%, and specificity of 70%). An increase in NT-proBNP of 1000 pg/mL elevated the risk of the presence of LGE by 20%, with adjustments for the left atria area, hemoglobin, white blood cell count, and LV mass.

### 3.4. Associations between ECV and Biomarkers

On examination of all the parameters associated with ECV, only cardiac-specific biomarkers—NT-proBNP and hs-TnT—along with atrial fibrillation were independently associated with ECV (Table 4). An NT-proBNP increase of 500 pg/mL was related to ECV elevation by 2.6%, along with an increase of hs-TnT of 1 ng/mL by 8.31%, with adjustments for age, NYHA class, presence of atrial fibrillation and ventricular arrhythmias, uric acid, and cholesterol LDL.

## 4. Discussion

### 4.1. Rationale of Circulating Markers of Fibrosis

Following cardiac injury (e.g., myocardial infarction, myocarditis, etc.) the whole heart is a subject to unprecedented stress, which activates compensatory mechanisms, including systemic activation of the neuro-hormonal axis, as well as local responses, resulting in myocyte hypertrophy and fibrosis. The critical feature of cardiac fibrosis is the trans-differentiation of fibroblasts into myofibroblasts, which is dependent on adrenergic and renin-angiotensin-aldosterone systems, as well as profibrotic cytokines, growth factors, and microRNAs [1,16,17,18]. Activated myofibroblasts secrete excessive amounts of extracellular procollagen chains into the interstitium, which assemble into fibrils and are cross-linked by lysyl oxidase [1,2]. Surrounding cardiomyocytes and myofibroblasts release pro-fibrotic cytokines and growth factors, including TGF, CTGF, PDGF, endothelin, interleukins, and angiotensin II, via paracrine mechanisms, which maintain the ‘pro-fibrotic milieu’ and attract monocytes and macrophages that further remodel the ECM. Unsurprisingly, myocardial concentrations of fibrosis-involved molecules, which can be measured via immunohistochemistry or polymerase chain reaction in cardiac samples harvested during EMB, are correlated with invasively-determined cardiac fibrosis [19]. These observations emerged from research into the potential role of circulating counterparts of fibrosis-related molecules as easily-accessible markers of cardiac fibrosis [11,19]. Due to the complexity of cardiac fibrosis pathology and its related pathways, several clusters of fibrosis-related molecules have been identified, such as markers of collagen synthesis and degradation (PICP, PIIINP, and CTPI); cytokines and growth factors (TGF-β, CTGF, PDGF-β, GDF-15, cardiotrophin-1, galectin-3, sST-2, osteopontin, or fibrosis-specific microRNAs); and ECM proteolytic enzymes—matrix metalloproteinases (MMPs) and their tissue inhibitors (TIMPs) [6,11]. Despite some initial enthusiasm, it transpires that the behavior of circulating fibrosis-related molecules may not parallel findings obtained in studies of histologically proven cardiac fibrosis [6,11].

### 4.2. Invasive vs. Non-Invasive Assessments of Cardiac Fibrosis

The method applied in the course of cardiac fibrosis assessment is of paramount importance. Biopsies can be qualitative (i.e., detecting the presence or absence of fibrosis) or quantitative, expressed as the collagen volume fraction (CVF) assessment of cardiac fibrosis [9,20]. Intuitively, an invasive diagnosis of fibrosis may be preferable over non-invasive methods, representing a more accurate means of investigation. On the other hand, CMR allows not only for both the qualitative and quantitative assessment of cardiac fibrosis, but also for the discrimination between replacement and interstitial fibrosis [10]. In fact, CMR is the much-preferred contemporary method of assessment for cardiac fibrosis given the numerous limitations of EBM, e.g., procedure-associated risks, the limited representation of the entire heart, sampling errors, the sampling of only superficial endocardial and myocardial layers, the high intra- and inter-observer variability of sample assessments, and last but not least, the highly-questionable redo-EBM for fibrosis monitoring [3,8,9]. Referral studies have shown a correspondence between EMB-determined CVF and CMR-assessed LGE or ECV; nevertheless, CMR-based parameters do not simply mirror EMB parameters [10,21]. Instead, CMR assessment allows for the more thorough inspection of the whole myocardium, including the mid-myocardium or epicardium, areas that are beyond the reach of EMB studies.

One of the advantages of this study is the simultaneous, quantitative assessment of both types of cardiac fibrosis in a homogenous group of 100 DCM patients. Consistently with the work of other authors, we observed a weak correlation between indices of replacement and interstitial fibrosis [21,22]. This observation merely highlights the fact that replacement and interstitial fibroses are distinct entities with differing pathological backgrounds and meanings, and thus should not be viewed as one ‘unit’. This is very well reflected in the study population’s baseline characteristics, including clinical, echocardiographic, and laboratory parameters, which revealed a lack of commonality between patients grouped according to LGE (absent/present) or ECV (lower/upper median).

### 4.3. Relationship between Circulating Fibrosis-Related Molecules and EMB-Assessed Cardiac Fibrosis

In earlier studies, Querejeta et al. and Izawa et al. reported associations between markers of collagen synthesis—PICP and PIIINP—with biopsy-proven fibrosis in HF patients [23,24]. In more recent studies, Yang et al. observed correlations between PICP and MMP-2 levels with CVF in hypertrophic cardiomyopathy (HCM) patients, whereas Ravassa et al. showed that the ratio of CITP/MMP-1 and PICP were associated with biopsy-proven fibrosis in hypertensive HF patients [25,26]. On the other hand, there is an abundance of studies reporting a dearth of associations between circulating molecules and EMB-assessed fibrosis. We reported that 12 serum markers of ECM metabolism, such as markers of collagen synthesis and controlling factors, including galectin-3, as well as the MMPs/TIMPs system, were of zero utility when it came to the prediction of EBM-assessed fibrosis in DCM [6,27]. Similarly, Du et al. reported no association between galectin-3, GDF-15, and TIMP-1 and biopsy-determined cardiac fibrosis in hypertensive HF [28]. Summarizing the current knowledge on the interactions of circulating molecules and EBM-determined fibrosis, Lopez et al. reported that out of 28 molecules studied, it was only markers of collagen synthesis and degradation (PIINP and CITP) that were clearly related to cardiac fibrosis, whereas for the majority of biomarkers, the results were weak, inconclusive, or even devoid of any relations [11]. Intriguingly, there are reports that showed clear associations between circulating fibrosis-linked microRNAs—miR-26 and miR-30—with CVF in DCM [29].

### 4.4. Relationship between Circulating Fibrosis-Related Molecules and CMR-Assessed Cardiac Fibrosis 

The few studies that have explored associations between circulating molecules and replacement fibrosis have also supplied puzzling results. Furthermore, their interpretation is all the more challenging due to the fact that heterogeneous populations with various degrees of fibrosis were investigated. On the one hand, there have been studies on DCM, HCM, and patients with CAD that showed correlations between galectin-3 and MMP-9 with LGE [30,31,32]. Conversely, studies on HCM, which explored TGF-β1; MMP-2 and MMP-9; TIMP-1; galectin-3; sST2; CITP, PICP, and PIIINP; hypertensive HF with PICP, PIIINP, CITP and MMP-1; valvular diseases (MMP-2, TIMP-1); and acute myocardial infraction populations (sST2) have reported a lack of any relationship between the biomarkers under analysis and LGE [33,34,35,36].

There are even fewer studies on the relationship between circulating markers and interstitial fibrosis and, in fact, most of these have reported no associations. In HCM, markers of collagen type I and III synthesis (PINP and PIIINP) were related neither to LGE extent nor to post-contrast T1 time [37]. Similarly, Foussier et al. who studied the MMP-2/TIMP-1 system in patients with valvular diseases, showed a very weak relationship between MMP-2 and TIMP-1 and ECV [35]. In line with this, Lepojärvi et al. also found no relations between PINP, PIIINP, and ST2 serum levels with post-contrast T1 times in CAD [32]. Interestingly, Fang et al. reported correlations between 10 microRNAs (miR-18, miR-146, miR-30, miR-17, miR-200, miR-19, miR-21, miR-193, miR-10, miR-15) and post-contrast T1 time in HCM, whereas PINP and PIIINP were consistently unrelated to interstitial fibrosis [38]. Hence, in agreement with the majority of studies published to date, we too did not observe any relationship between markers of collagen type I and III synthesis and degradation (PICP, PIIINP, CTIP) and fibrosis-controlling factors (CT-1, GDF-15 and PDGF) with replacement or interstitial fibrosis in our large and contemporary cohort of DCM patients.

In endeavoring to account for these ‘negative’ findings, all of the following shortcomings and limitations of biomarker-based organ fibrosis assessment should be acknowledged: insufficiently organ-specific markers (potential diagnostic noise in the case of concomitant fibrotic diseases, e.g., cardiac fibrosis with liver or kidney fibrosis, neoplasms, etc.), unspecific abnormalities of marker blood levels in acute or chronic infections, low-grade chronic inflammation (connective tissue diseases, atherosclerosis), and/or tissue- and wound-healing following injuries [11,19,20,22]. Finally, for the sake of argument—in order to be pathology-relevant and clinically useful—the ideal ‘circulating marker of cardiac fibrosis’ should reflect differing types of fibrosis, e.g., replacement vs. interstitial fibrosis. One might theorize that one marker should be related to replacement and the other to interstitial fibrosis in order to be sure which process is actually being investigated. Alternatively, significantly dissimilar cut-off values (i.e., different values for replacement and interstitial fibrosis) of the same marker should be used. Obviously, our results can be viewed, and perhaps interpreted, as ‘negative’, but this would be only partially true. On the contrary, we believe that we provide rather solid evidence regarding the lack of association between various fibrosis-linked circulating molecules (some of which were previously found to be related with biopsy-determined fibrosis) with replacement and interstitial fibrosis in one of the largest DCM cohorts. Although the elucidation of these issues is still far from being complete, we nevertheless provide another argument for the lack of utility of ‘fibrosis-related’ molecules and CMR-assessed cardiac fibrosis, at least in DCM, and perhaps in younger HF populations.

### 4.5. Relationship between Cardiac-Specific Biomarkers and Replacement and Interstitial Fibrosis

In contrast to fibrosis-related markers, which are not organ-specific but rather reflect an on-going systemic process, two highly-specific cardiac biomarkers—NT-proBNP and hs-TnT—were indeed strongly related to both replacement and interstitial fibrosis in our DCM cohort. Although studied in different populations, similar findings have been reported by other authors. Kawasaki et al. reported associations between BNP and hs-TnT with LGE in HCM patients [39]. In addition, Ho et al. found correlations between NT-proBNP and ECV in HCM patients, both with and without mutations in sarcomeric genes [40]. In patients after acute ST-elevation myocardial infraction, LGE extent was highly correlated with hs-TnT [41]. The first to address this issue specifically in DCM were Karaahmet et al., who reported significant differences in NT-proBNP levels in 40 DCM patients with and without LGE, and a strong correlation with the degree of LGE [42]. Furthermore, there are a few smaller studies (i.e., with fewer than 40 patients) that have explored the link between NT-proBNP and interstitial fibrosis in DCM. Yoon et al. observed in 24 DCM patients that NT-proBNP was negatively correlated with post-contrast T1 time [43]. Similarly, Tachi et al. and Child et al. reported correlations between post-contrast T1 times and BNP in DCM [44,45]. In the same vein, Child et al. and Mazurkiewicz et al. showed associations between ECV and NT-proBNP [45,46].

Evidence for an association between troponin and interstitial fibrosis in HF and DCM is scant. We did not observe any link between hs-TnT and replacement fibrosis (i.e., LGE extent), which is in agreement with previous research from Yi et al. [47]. However, we detected a strong association (HR 8.3) between hs-TnT and interstitial fibrosis (i.e., ECV), which is similar to the observations of Mazurkiewicz et al., who also revealed a link between hs-TnT and ECV in DCM [46].

Although the causality of the observed link between cardiac-specific biomarkers (NT-proBNP and hs-TnT) and cardiac fibrosis has not been clearly established, a basic line of reasoning that may contribute to an understanding of the abovementioned results can be tentatively developed. As the LV progressively dilates, wall tension on the LV walls increases, which in turn triggers NT-proBNP production and release. The fibrotic myocardium, which is less compliant than normal LV walls, disproportionally increases tension on the remaining cardiomyocytes, contributing to the release of even more NT-proBNP. Likewise, more stressed and damaged cardiomyocytes release a larger amount of troponins, which are markers of myocardial injury. Thus, the observations presented here concerning the clear link between cardiac-specific biomarkers of myocardial stress (NT-proBNP) and injury (hs-TnT) with cardiac fibrosis arise from the pathology of the cardiac remodeling process typically seen in HF and DCM.

### 4.6. Study Limitations

We acknowledge several limitations of the study. Firstly, although this is one of the largest published studies of this kind, it is still a single-center study with a relatively small number of patients. Secondly, the blood kinetics of fibrosis-related molecules may depend on the blood volume status, which is particularly relevant to HF populations. We tried to correct for this and recruited only patients in stable conditions and not requiring intravenous diuretic or fluid therapy in cases of hyper- or hypovolemia. In addition, concentrations of blood markers may vary between day-to-day or week-to-week measurements. Perhaps, blood sampling over a longer period may provide a more meaningful mean concentration of those markers. Although it is feasible to perform CMR examinations in patients with some approved cardiac devices, such as pacemakers, implantable cardioverter-defibrillators, etc.; nevertheless, the quality of CMR scans, including T1-mapping, is profoundly impaired, making the assessment of some cardiac segments virtually impossible. Therefore, we decided not to include DCM patients with cardiac devices in the present study.

## 5. Conclusions

None of the circulating fibrosis markers, including markers of collagen type I and III synthesis and degradation, differentiated between DCM patients with and without replacement fibrosis, or patients stratified according to whether they were lower- or upper-median in terms of ECV. However, it was seen that cardiac-specific markers NT-proBNP and hs-TnT were significantly lower in patients without LGE or in the lower ECV strata, whereas NT-proBNP and hs-TnT were elevated in patients with LGE and in the upper ECV strata. NT-proBNP levels, along with LV mass and white blood count, were found to be independently associated with replacement fibrosis, whereas NT-proBNP and hsTnT, as well as atrial fibrillation, were independently associated with interstitial fibrosis. This study provides strong evidence for the lack of a relationship between circulating markers of fibrosis and the CMR-based quantitative assessment of replacement and interstitial fibrosis in a contemporary homogenous cohort of DCM patients. Levels of circulating markers of fibrosis seem to be of no utility in the diagnosis and monitoring of cardiac fibrosis in DCM.

## Figures and Tables

**Table 1:** Baseline characteristics. Comparison of DCM patients with and without LGE and DCM patients stratified according to median value of ECV.

	Without LGE (*n* = 56)	With LGE (*n* = 44)	*p*-Value	ECV ≤ Median (*n* = 50)	ECV > Median (*n* = 50)	*p*-Value
Age (year)	44.86 ± 11.28	45.42 ± 12.29	0.76	41.87 ± 11.13	50.06 ± 11.06	0.0015
Male (*n*, %)	49 (87.5%)	38 (86.4%)	0.87	40 (80%)	45 (90%)	0.13
BMI (kg/m^2^)	28.21 ± 5.58	28.88 ± 5.97	0.54	29.08 ± 5.75	28.2 ± 6.13	0.50
HF symptoms time (month)	18.69 ± 24.73	12.84 ± 21.7	0.32	13.75 ± 19.15	16.07 ± 27.26	0.41
Diabetes mellitus (*n*, %)	7 (12.5%)	7 (15.9%)	0.63	7 (14%)	8 (16%)	0.76
Hypercholesterolemia (*n*, %)	31 (55.4%)	30 (68.2%)	0.19	31 (62%)	28 (56%)	0.66
Hypertension (*n*, %)	5 (8.9%)	15 (34.1%)	0.002	9 (18%)	11 (22%)	0.60
COPD (*n*, %)	1 (1.8%)	5 (11.4%)	0.045	1 (2%)	6 (12%)	0.09
Atrial fibrillation (*n*, %)	12 (21.4%)	14 (31.8%)	0.24	7 (14%)	22 (44%)	0.02
NYHA class	1.73 ± 0.6	1.88 ± 0.63	0.26	1.72 ± 0.56	1.97 ± 0.65	0.10
SBP (mmHg)	118.9 ± 17.3	121.8 ± 21.8	0.46	121.2 ± 16.8	118.6 ± 22.1	0.53
DBP (mmHg)	75.16 ± 12.35	80.3 ± 14.25	0.03	76.68 ± 11.89	78.52 ± 14.24	0.51
Heart rate (bpm)	70.54 ± 12.69	72.72 ± 14.71	0.74	69.81 ± 15.11	74.58 ± 12.98	0.12
QRS (ms)	95.64 ± 24.17	106.14 ± 29.9	0.02	100.23 ± 28.07	102.05 ± 28.58	0.63
Ventricular arrhythmia (*n*, %)	17 (31.5%)	14 (32.6%)	0.91	7 (14%)	26 (52%)	<0.001
6MWT—distance (m)	451.6 ± 90.4	438.7 ± 94.5	0.49	464.6 ± 89.2	416.1 ± 92.5	0.01
LVEDd/BSA (mm/m^2^)	32.03 ± 4.51	30.99 ± 5.16	0.16	31.26 ± 4.35	31.85 ± 5.6	0.58
IVS (mm)	9.65 ± 1.92	10.52 ± 2.47	0.05	9.66 ± 1.84	10.43 ± 2.58	0.11
LVEF (%)	30.88 ± 10.14	28.06 ± 9.98	0.17	31.22 ± 9.89	27.47 ± 11.15	0.10
RVd (mm/m^2^)	39.3 ± 6.4	39.34 ± 7.21	0.90	38.48 ± 6.58	40.52 ± 7.25	0.17
LAA (cm^2^)	26.24 ± 6.79	29.98 ± 9.62	0.05	25.81 ± 8.2	30.98 ± 8.01	<0.001
RAA (cm^2^)	20.16 ± 6.79	21.69 ± 6.24	0.10	19.07 ± 5.26	23.4 ± 7.44	0.001
E/e’	9.17 ± 5.69	12.23 ± 5.77	0.003	9.96 ± 5.9	11.54 ± 6.33	0.25
MR ≥ moderate (*n*, %)	17 (30.4%)	17 (38.6%)	0.39	9 (18%)	27 (54%)	<0.001
TR ≥ moderate (*n*, %)	7 (12.5%)	4 (9.1%)	0.59	2 (4%)	9 (18%)	0.04
TRV (m/s)	2.58 ± 1.39	2.32 ± 1.23	0.37	2.32 ± 1.23	2.74 ± 1.44	0.14
LV mass (g)	165.4 ± 44.2	203.5 ± 53.2	<0.001	172.7 ± 48.3	193.2 ± 55.3	0.13
% LGE (%)	0	4.55 ± 5.02	<0.001	1.88 ± 1.59	5.85 ± 5.67	0.01
T1 native (ms)	1207 ± 188	1285.2 ± 64.2	0.009	1255.5 ± 104.9	1274.7 ± 145.1	<0.001
T1 post contrast (ms)	470.7 ± 53.6	471.3 ± 44.1	0.95	485.3 ± 49.4	458.2 ± 42.4	0.007
ECV (%)	28.0 ± 5.4	29.8 ± 4.2	0.01	25.3 ± 1.8	32.4 ± 4.5	<0.001
Hct (%)	42.26 ± 3.73	44.43 ± 5	0.01	43.49 ± 4.57	42.91 ± 4.6	0.55
WBC (M/uL)	6.96 ± 2.05	8.52 ± 1.99	<0.001	7.25 ± 2.09	8.1 ± 2.27	0.07
Creatinine (umol/L)	90.34 ± 41.34	92.7 ± 21.83	0.13	92.91 ± 46.57	90.77 ± 21	0.64
Uric acid (umol/L)	400.4 ± 112.2	450.4 ± 114.6	0.04	386.11 ± 102.29	455.51 ± 126.41	0.01
Cholesterol LDL (mmol/L)	3.14 ± 0.95	3 ± 0.85	0.45	3.29 ± 0.94	2.82 ± 0.81	0.01
BB (*n*, %)	56 (100%)	44 (100%)	1.00	50 (100%)	50 (100%)	1.00
ARNI/ACEi (*n*, %)	56 (100%)	43 (97.7%)	0.26	50 (100%)	49 (98%)	0.31
MRA (*n*, %)	55 (98.2%)	41 (93.2%)	0.20	48 (96.0%)	48 (96.0%)	1.00
Diuretic dosage (mg/day)	35.12 ± 73.31	46.5 ± 42.48	0.03	28.95 ± 34.98	58.69 ± 82.12	0.01

Abbreviations: LGE—late gadolinium enhancement, ECV—extracellular volume, BMI—body mass index, HF—heart failure, COPD—chronic obstructive pulmonary disease, NYHA—New York Heart Association class, SBP/DBP—systolic/diastolic blood pressure, 6MWT—6-min walk test, LVEDd—left ventricle end-diastolic diameter, BSA—body surface area, LVEF—left ventricle ejection fraction, LAA/RAA—left/right atria area, MR/TR—moderate or severe mitral/tricuspid regurgitation, TRV—TR peak velocity, LGE—late gadolinium enhancement, ECV—extracellular volume, LV—left ventricle, Hb—hemoglobin, Hct—hematocrit, BB—beta-blocker, ARNI—angiotensin receptor-neprilysin inhibitor, ACEI—angiotensin-converting-enzyme inhibitor, MRA—mineralocorticoid receptor antagonist.

**Table 2:** Comparison of biomarkers between DCM patients, stratified according to LGE and ECV.

	Without LGE (*n* = 56)	With LGE (*n* = 44)	*p*-Value	ECV ≤ Median (*n* = 50)	ECV > Median (*n* = 50)	*p*-Value
NT-proBNP (pg/mL)	345 (109.5–1047)	972 (432–1818)	<0.001	389 (81- 793.5)	1371 (750–2652)	<0.001
hsTnT (ng/mL)	0.007 (0.005–0.014)	0.013 (0.009–0.02)	<0.001	0.009 (0.007–0.018)	0.013 (0.007–0.02)	<0.001
CT-1 (pg/mL)	42.3 (6.1–169.2)	81.9 (4.9–225.1)	0.81	60.3 (6.1–189)	37.1 (4.9–225.1)	0.69
PDGF-BB (pg/mL)	254.7 (139.3–334.3)	215.4 (135–362)	0.36	221.6 (133–334)	216.8 (139–331)	0.95
GDF-15 (pg/mL)	26.5 (18.3–47.1)	28.3 (20.3–60.9)	0.67	24.9 (18.1–40.8)	33.7 (23.3–60.3)	0.08
PICP (ng/mL)	95.5 (65.7–173.4)	75.8 (68.4–204)	0.72	97.7 (71.7–306)	77.1 (65.7–146)	0.09
PIIINP (ng/L)	161.2 (127.9–350.5)	163.9 (135–445)	0.79	165.5 (129–571)	157 (127.9–309)	0.26
CTIP (ng/mL)	0.32 (0.22–0.38)	0.28 (0.22–0.34)	0.91	0.28 (0.21–0.35)	0.29 (0.23–0.35)	0.41

Abbreviations: NT-proBNP—N-terminal fragment of the prohormone B-type natriuretic peptide, hsTnT—high-sensitive troponin T, CT-1—cardiotrophin-1, GDF-15—growth/differentiation factor-15, PDGF-BB—platelet-derived growth factor subunit B, PICP—procollagen I C-terminal propeptide, PIIINP—procollagen III N-terminal propeptide, CTPI—terminal telopeptides of type I collagen (CTPI).

**Table 3:** Uni- and multivariate logistic regression models for the prediction of LGE presence.

	Univariate		Multivariate	
	OR (95%CI)	*p*-Value	OR (95%CI)	*p*-Value
QRS (ms)	1.015 (0.999–1.031)	0.06		
LAA (cm^2^)	1.058 (1.005–1.115)	0.03	0.973 (0.91–1.04)	0.42
Hb (g/dL)	1.322 (1.003–1.742)	0.04	1.112 (0.799–1.548)	0.52
WBC (M/uL)	1.451 (1.168–1.801)	<0.001	1.288 (0.997–1.664)	0.05
Uric acid (umol/L)	1.004 (0.999–1.008)	0.05		
hsTnT (ng/mL)	3 × 10^11^ (6 × 10^−3^–4 × 10^33^)	0.10		
log10 (NT-proBNP)	3.343 (1.619–6.901)	0.001	2.979 (1.216–7.296)	0.02
LV mass (g)	1.016 (1.007–1.026)	<0.001	1.016 (1.004–1.029)	0.009
loop diuretics (mg/d)	1.003 (0.996–1.010)	0.38		
QRS (ms)	1.015 (0.999–1.031)	0.06		

**Table 4:** Uni- and multivariate linear regression models for ECV.

	Univariate		Multivariate	
	Standard Coefficient	*p*-Value	Standard Coefficient	*p*-Value
Age (years)	0.13 ± 0.04	0.002	0.03 ± 0.04	0.45
NYHA class	2.37 ± 0.81	0.005	0.47 ± 0.87	0.59
Atrial fibrillation	−1.47 ± 0.56	0.01	−1.09 ± 0.55	0.05
Ventricular arrhythmias	−1.57 ± 0.53	0.004	−0.73 ± 0.53	0.17
COPD	−1.03 ± 1.03	0.32		
IVS (mm)	0.43 ± 0.23	0.07		
LAA (cm^2^)	0.09 ± 0.06	0.13		
Uric acid (umol/L)	0.011 ± 0.004	0.02	0.004 ± 0.004	0.31
Cholesterol LDL (mmol/L)	−1.56 ± 0.57	0.008	−0.97 ± 0.63	0.13
hsTnT (ng/mL)	11.92 ± 4.31	0.007	8.31 ± 3.70	0.03
log10 (NT-proBNP)	3.04 ± 0.67	<0.001	1.78 ± 0.80	0.03
hsCRP (mg/dL)	0.09 ± 0.06	0.13		
Loop diuretics (mg/day)	0.02 ± 0.01	0.06		

## Data Availability

Some data will be available on: https://www.szpitaljp2.krakow.pl, access on 23 May 2021.

## Supplementary Materials

The following are available online at https://www.mdpi.com/article/10.3390/cells10061295/s1, Table S1: Comparison of baseline characteristics and circulating markers of fibrosis between DCM patients and control group.
