# Supplementary material for: Lack of Relationship between Fibrosis-Related Biomarkers and Cardiac Magnetic Resonance-Assessed Replacement and Interstitial Fibrosis in Dilated Cardiomyopathy
Source: Cells. 2021 May 23;10(6):1295. doi: 10.3390/cells10061295 (PMC8224556; doi:10.3390/cells10061295)
Supplement: Supplementary file 1 [file cells-10-01295-s001.zip › cells-1221382-supplementary.pdf]

Table S1. Comparison of baseline characteristics and circulating markers of fibrosis between DCM patients and control group.

Parameter	DCM patients (n=100)	Control group (n=27)	p-value
Age [years]	45.2 ± 11.8	40.9 ± 12.3	0.24
Male sex (n, %]	87 (87)	21 (77.8)	0.79
BMI [kg/m^2]	28.5 ± 5.7	24.83 ± 3.75	0.05
LVEDd [mm]	63.8 ± 8.2	45.00 ± 7.24	<0.001
EF [%]	29.7 ± 10.1	58.60 ± 2.51	<0.001
Cardiotrophin-1 [pg/ml]	138.9 ± 194.3	38.1 ± 21.1	<0.001
PDGF-BB [pg/ml]	363.7 ± 453.6	87.4 ± 37.9	<0.001
GDF-15 [pg/ml]	38.9 ± 41.1	17.8 ± 11.6	<0.01
PICP [ng/ml]	203.5 ± 260.5	55.1± 50.8	<0.003
PIIINP [ng/L]	366.6 ± 412.1	96.3 ± 15.9	<0.01
CTX-I [ng/ml]	0.31 ± 0.12	0.16 ± 0.12	<0.001
